# Supplementary material for: Assessing fish welfare in small-scale commercial fixed-net fisheries off the Southern Portuguese coast
Source: PLoS One. 2025 Dec 18;20(12):e0330004. doi: 10.1371/journal.pone.0330004 (PMC12714217; doi:10.1371/journal.pone.0330004)
Supplement: S1 Table — (DV: Two- banded seabream (Diplodus vulgaris), MS: Red mullet (Mullus surmuletus), PA: Axillary seabream (Pagellus acarne), PE: Common pandora (Pagellus erythrinus). (PDF) [file pone.0330004.s001.pdf]

S1 Table: The Estimate, standard error, and the p- values (*p*: Pr(>|z|)) derived from the Generalised linear models (GLMs) that were fit to predict the impact of several biological, operational, and environmental predictors on the duration of fish activity on the deck of the fishing vessel. (DV: Two- banded seabream (*Diplodus vulgaris*), MS: Red mullet (*Mullus surmuletus*), PA: Axillary seabream (*Pagellus acarne*), PE: Common pandora (*Pagellus erythrinus*)

	**DV**			**MS**			**PA**			**PE**		
Predictors	*Intercept*	*Std. Error*	*p*	*Intercept*	*Std. Error*	*p*	*Intercept*	*Std. Error*	*p*	*Intercept*	*Std. Error*	*p*
Intercept	4.66	0.449	**<0.001**	3.784	1.14	**<0.001**	2.361	0.564	**<0.001**	3.052	0.226	**<0.001**
Scale loss [2]												
Mesh Size [78 mm]							0.891	0.087	**<0.001**			
Depth (m)	-0.011	0.002	**<0.001**									
Fishing depth temperature (°C)				0.177	0.067	**<0.01**	-0.236	0.0432	**<0.001**			

Sea surface temperature (°C)							0.241	0.036	**<0.001**			
Atmospheric temperature (°C)	-0.09	0.018	**<0.001**				-0.055	0.018	**<0.01**	-0.052	0.011	**<0.001**
Observations (n)	100			102			100			101		
