# Supplementary material for: Assessing fish welfare in small-scale commercial fixed-net fisheries off the Southern Portuguese coast
Source: PLoS One. 2025 Dec 18;20(12):e0330004. doi: 10.1371/journal.pone.0330004 (PMC12714217; doi:10.1371/journal.pone.0330004)
Supplement: S2 Table — (1. All species: The full model consisting of all the species, DV: Two- banded seabream (Diplodus vulgaris), MS: Red mullet (Mullus surmuletus), PA: Axillary seabream (Pagellus acarne); Vitality scale: 4 = highly active, 3 = less active, 2 = lethargic and 1 = unresponsive). (PDF) [file pone.0330004.s002.pdf]

S2 Table: The Odds ratios, CI: Confidence intervals, and the p- values derived from the Cumulative linked mixed models (CLMMs) that were fit to predict the impact of several biological, operational, and environmental predictors on the vitality at the time of landing on deck. (1. All species: The full model consisting of all the species, DV: Two- banded seabream (*Diplodus vulgaris*), MS: Red mullet (*Mullus surmuletus*), PA: Axillary seabream (*Pagellus acarne*); Vitality scale: 4 = highly active, 3 = less active, 2 = lethargic and 1 = unresponsive)

	**All species**			**DV**			**MS**			**PA**		
Predictors	*Odds Ratios*	*CI*	*p*	*Odds Ratios*	*CI*	*p*	*Odds Ratios*	*CI*	*p*	*Odds Ratios*	*CI*	*p*
1 | 2	0.05	0.03-0.11	**<0.001**	0.01	0.00-0.04	**<0.001**	1.08	0.43-2.75	0.865	0.12	0.05-0.27	**<0.001**
2 | 3	0.21	0.11-0.39	**<0.001**	0.08	0.03-0.25	**<0.001**	4.32	1.60-11.67	**0.004**	0.67	0.37-1.21	0.182
3 | 4	0.77	0.42-1.42	0.4	1.05	0.44-2.53	0.905	8.79	3.10-24.95	**<0.001**	1.49	0.85-2.61	0.16
Species [MS]	0.08	0.04-0.17	**<0.001**									
Species [PA]	1.01	0.51-2.01	0.97									
Species [PE]	1.68	0.85-3.32	0.138									
Scale loss [2]	0.27	0.11-0.65	**0.003**	0.02		**0.039**	0.36		0.077			
Length (cm)							0.52		**0.028**			
Mesh size [78mm]							7.54		0.137	20.02	4.71-85.06	**<0.001**
Fishing depth temperature (°C)							0.4		**0.033**			
Sea surface temperature (°C)				2.2	1.03-4.69	**0.042**	0.0			2.6	1.53-4.41	**<0.001**
Observations (n)	403			100			100			102		
