# Supplementary material for: Assessing fish welfare in small-scale commercial fixed-net fisheries off the Southern Portuguese coast
Source: PLoS One. 2025 Dec 18;20(12):e0330004. doi: 10.1371/journal.pone.0330004 (PMC12714217; doi:10.1371/journal.pone.0330004)
Supplement: S3 Table — (DV: Two- banded seabream (Diplodus vulgaris), PA: Axillary seabream (Pagellus acarne), PE: Common pandora (Pagellus erythrinus); Anova: Analysis of variance, KW: Kruskal- Wallis test, t-test: Welch two sample t-test). (PDF) [file pone.0330004.s003.pdf]

**S3 Table: The p-values obtained from statistical significance tests performed to compare the levels of physiological stress parameters between vitality stages depending upon the vitality at arrival.** (DV: Two- banded seabream (*Diplodus vulgaris*), PA: Axillary seabream (*Pagellus acarne*), PE: Common pandora (*Pagellus erythrinus*); Anova: Analysis of variance, KW: Kruskal- Wallis test, t-test: Welch two sample t-test)

| Species | Vitality at arrival | Physiological stress parameter | p-value |
|---|---|---|---|
| **DV** | 4 (Between vitality stages 4.4, 4.3, 4.2, 4.1) | Cortisol (ng/ml) | KW- 0.483 |
| | | Glucose (mM) | Anova- 0.634 |
| | | Lactate (mM) | Anova- 0.653 |
| | | Osmolality (mOsm/Kg) | Anova- 0.2459 |
| | 3 (Between vitality stages 3.3, 3.2, 3.1) | Cortisol (ng/ml) | Anova- 0.0730 |
| | | Glucose (mM) | Anova- 0.7645 |
| | | Lactate (mM) | Anova- 0.229 |
| | | Osmolality (mOsm/Kg) | Anova- 0.252 |
| | 2 (Between vitality stages 2.2, 2.1) | Cortisol (ng/ml) | t- test- 0.577 |
| | | Glucose (mM) | t- test- 0.573 |
| | | Lactate (mM) | t- test- 0.385 |
| | | Osmolality (mOsm/Kg) | t- test- 0.126 |
| **PA** | 4 (Between vitality stages 4.4, 4.3, 4.2, 4.1) | Cortisol (ng/ml) | KW- 0.463 |
| | | Glucose (mM) | Anova- 0.344 |
| | | Lactate (mM) | Anova- 0.908 |
| | | Osmolality (mOsm/Kg) | Anova- 0.927 |
| | 3 (Between vitality stages 3.3, 3.2, 3.1) | Cortisol (ng/ml) | Anova- 0.516 |
| | | Glucose (mM) | KW- 0.456 |
| | | Lactate (mM) | Anova- 0.305 |
| | | Osmolality (mOsm/Kg) | Anova- 0.176 |
| | 2 (Between vitality stages 2.2, 2.1) | Cortisol (ng/ml) | t-test- 0.347 |
| | | Glucose (mM) | t-test- 0.509 |
| | | Lactate (mM) | t-test- 0.995 |
| | | Osmolality (mOsm/Kg) | t-test- 0.537 |
| **PE** | 4 (Between vitality stages 4.4, 4.3, 4.2, 4.1) | Cortisol (ng/ml) | Anova- 0.2166 |
| | | Glucose (mM) | Anova- 0.0689 |
| | | Lactate (mM) | Anova- 0.649 |
| | | Osmolality (mOsm/Kg) | Anova- 0.369 |
| | 3 (Between vitality stages 3.3, 3.2, 3.1) | Cortisol (ng/ml) | KW- 0.455 |
| | | Glucose (mM) | Anova- 0.612 |
| | | Lactate (mM) | Anova- 0.378 |
| | | Osmolality (mOsm/Kg) | Anova- 0.474 |
| | 2 (Between vitality stages 2.2, 2.1) | Cortisol (ng/ml) | t- test- 0.242 |
| | | Glucose (mM) | t- test- 0.997 |
| | | Lactate (mM) | t- test- 0.529 |

| | Osmolality (mOsm/Kg) | t- test- 0.083 |
|---|---|---|
