# Supplementary material for: Assessing fish welfare in small-scale commercial fixed-net fisheries off the Southern Portuguese coast
Source: PLoS One. 2025 Dec 18;20(12):e0330004. doi: 10.1371/journal.pone.0330004 (PMC12714217; doi:10.1371/journal.pone.0330004)
Supplement: S4 Table — (DV: Two- banded seabream (Diplodus vulgaris), PA: Axillary seabream (Pagellus acarne), PE: Common pandora (Pagellus erythrinus); Anova: Analysis of variance, KW: Kruskal- Wallis test). (PDF) [file pone.0330004.s004.pdf]

S4 Table: The p-values obtained from statistical significance tests performed to compare the levels of physiological stress parameters in vitality stages where the vitality at arrival was the same as vitality at sampling (stages 4.4, 3.3, 2.2, and 1.1) as indicated by the striped bars in Fig 7. (DV: Two- banded seabream (*Diplodus vulgaris*), PA: Axillary seabream (*Pagellus acarne*), PE: Common pandora (*Pagellus erythrinus*); Anova: Analysis of variance, KW: Kruskal- Wallis test).

Species	Physiological stress parameter	*p- value*
**DV**	Cortisol (ng/ml)	*Anova- 0.562*
	Glucose (mM)	*Anova- 0.894*
	Lactate (mM)	*Anova- 0.252*
	Osmolality (mOsm/Kg)	*Anova- 0.713*
**PA**	Cortisol (ng/ml)	*Anova- 0.626*
	Glucose (mM)	*Anova- 0.237*
	Lactate (mM)	*Anova- 0.482*
	Osmolality (mOsm/Kg)	*Anova- 0.986*
**PE**	Cortisol (ng/ml)	*KW- 0.401*
	Glucose (mM)	*Anova- 0.764*
	Lactate (mM)	*Anova- 0.177*
	Osmolality (mOsm/Kg)	*Anova- 0.098*
